# Exploring the Utility of Urinary Creatinine Adjustment for KIM-1, NGAL, and Cystatin C for the Assessment of Kidney Function: Insights from the C-KidnEES Cohort

**DOI:** 10.3390/children11010015

**Published:** 2023-12-22

**Authors:** T. D. K. S. C. Gunasekara, Chula Herath, P. Mangala C. S. De Silva, Nishad Jayasundara

**Affiliations:** 1 Department of Zoology, Faculty of Science, University of Ruhuna, Matara 81000, Sri Lanka; gunasekara.sc166@fgs.ruh.ac.lk; 2 Department of Nephrology, Sri Jayewardenepura General Hospital, Colombo 10100, Sri Lanka; chulaherath@gmail.com; 3 Nicholas School of the Environment, Duke University, Durham, NC 27708, USA; nishad.jayasundara@duke.edu

**Keywords:** biomarkers, creatinine, cystatin C, KIM-1, NGAL, normalization

## Abstract

Normalization of urinary biomarkers of kidney injury is a common practice in clinical and research settings to account for variations in urine concentration, and urinary creatinine is often used as a reference. However, to date, there is no consensus on the adjustment of urinary biomarkers with creatinine, and both absolute and creatinine-adjusted biomarker levels are adopted for making interpretations of kidney health. Hence, the present study aimed to investigate the associations of urinary creatinine with three widely used kidney injury biomarkers, KIM-1, NGAL, and cystatin C, to validate the applicability of urinary creatinine as a reference for normalization. A cross-sectional study was performed with 2100 students, 10–18 years of age in the Children’s Kidney Environmental Exposure Study (C-KidnEES) cohort established in Sri Lanka. As identified in linear regression analyses, normalization of urinary KIM-1, NGAL, and Cys-C to urinary creatinine did not result in significant under-adjustment or over-adjustment to the absolute urinary concentrations, giving no specific rationale for creatinine adjustment. Hence, absolute urinary concentrations of the above biomarkers can be adopted for the characterization of subclinical kidney injury in adolescents in community studies where early morning urine sampling is practiced. However, for spot urine samples, consideration of both absolute and creatinine-adjusted biomarker levels would be a better approach.

## 1. Introduction 

Renal biomarkers resemble a wide spectrum of measurable molecules in urine and blood that play a pivotal role in the diagnosis of kidney diseases, monitoring kidney function, and early detection of kidney disease susceptibilities in community screening. Both urinary and serum markers have their own strengths and limitations. Hence, the combined use of multiple biomarkers offers a more comprehensive picture of kidney function and health. Urinary biomarkers are particularly advantageous compared to serum markers in many aspects. Mainly, urine samples can be easily collected through non-invasive methods, such as voided urine samples, which are less discomforting for patients compared to blood draws. This can lead to better patient compliance with monitoring protocols [1]. Urinary biomarkers are often directly produced by the kidneys or are filtered through the kidneys from the blood. This proximity to kidney tissues can make urinary biomarkers more sensitive to early changes in kidney function and damage. Urinary biomarker levels can change more rapidly in response to acute changes in kidney function before detectable changes in serum markers like creatinine. This is particularly important for identifying acute kidney injury (AKI) in its early stages. Importantly, urinary biomarkers can help distinguish between glomerular dysfunction (related to filtration) and tubular dysfunction (related to reabsorption and secretion). This differentiation is important for diagnosing differential renal pathologies. This enhanced specificity and sensitivity becomes highly important in detecting kidney damage or dysfunction early through urinary biomarkers, which can lead to timely interventions that might prevent further damage and improve patient outcomes [2]. 

In particular, compared to the conventional serum-based markers (e.g., serum creatinine, blood urea nitrogen, and estimated glomerular filtration rate), recently identified proteomic biomarkers such as kidney injury molecule-1 (KIM-1) and neutrophil gelatinase-associated lipocalin (NGAL) are known to enhance sensitivity and specificity, particularly in early stages of kidney diseases where the conventional markers show no detectable changes [3,4]. In community screening for kidney diseases, these novel markers provide early and specific insights into kidney injury, guiding interventions and improving the overall management of kidney diseases [5].

The use of spot urine samples for proteomic assays and biomarker testing is gaining increasing attention from researchers over 24 h urine collection as a more convenient diagnostic approach [6]. However, urine concentration is a dynamic process influenced by multiple factors including sex, age, muscle mass, diet [7], physical activity [8], time of the day, hydration status, medications, and kidney function [9,10]. Concentrated urine may lead to overestimation of biomarker concentrations, while diluted urine may lead to underestimation. By adjusting kidney injury biomarker concentrations to a reference, researchers can account for these dilution-related variations and compare biomarker levels more accurately across different individuals or time points. The choice of normalization method depends on the specific goals of the study, the nature of the biomarker being measured, and the availability of resources. At this point, urinary creatinine and specific gravity are known to be feasible references for the normalization of urinary biomarkers. Measuring creatinine is a relatively simple and cost-effective process, making it widely available and practical for clinical and research purposes. Specific gravity measurements, on the other hand, may require more specialized equipment, and the analysis is relatively time-consuming. Hence, urinary creatinine is often used as a reference marker to normalize the concentration of other urinary biomarkers, particularly in studies related to kidney function and injury [11].

Creatinine is a waste product generated by the breakdown of creatine phosphate in muscle tissue as a result of normal muscle metabolism. Once creatinine is formed in the muscles, it enters the bloodstream, and the clearance of creatinine is primarily handled by the kidneys. Since creatinine is excreted in urine at a relatively constant rate (assuming stable kidney function), it is often used as a marker to assess kidney function and to normalize the concentration of other substances in urine, including various biomarkers, for research and clinical purposes. The excretion of creatinine is governed by glomerular filtration and active tubular secretion. Also, the concentration of creatinine in the urine can vary depending on several factors such as age, sex, muscle mass, and kidney function [12]. Hence, the adjustment of urinary biomarker concentrations to creatinine is debated particularly in certain nephropathies such as acute kidney injury (AKI) [11]. However, researchers have adopted absolute and creatinine-adjusted biomarker levels for making interpretations of kidney health, and there exists no consensus for adjustment. Moreover, mass screening particularly with robust urinary markers is of utmost importance in the management of aggressive nephropathies such as chronic kidney disease of uncertain etiology (CKDu). However, the analysis of biospecimens is highly challenging for low- and low–middle-income countries. Hence, a better understanding of biomarker normalization and optimization of biomarker testing leads to significant savings in analytical costs. In this perspective, the present study examines the associations between urinary creatinine and three widely used kidney injury biomarkers, KIM-1, NGAL, and cystatin C, in healthy pediatric subjects, in order to validate the applicability of urinary biomarker adjustment to creatinine.

## 2. Materials and Methods

### 2.1. Study Communities

This cross-sectional study was conducted with school students (10–18 years of age) in the Children’s Kidney Environmental Exposure Study (C-KidnEES) cohort established in Sri Lanka. We encouraged voluntary participation of the students under the informed consent of the parents and the assent of the students. Following analysis of all samples, biomarker data of the students who had been diagnosed with kidney, metabolic, respiratory, and other persistent diseases, were undergoing medical treatments, and had regular sports practices were excluded from the analysis. In adherence to the above exclusion criteria, biomarker data of 2100 eligible candidates were selected for analysis out of the 2250 total participants in the study.

### 2.2. Data and Samples

Key demographic data along with information on current health status, persistent diseases, and medications were gathered using an interviewer-administered structured questionnaire. An on-site medical examination was conducted along with inspection of any available clinical records to investigate the medical history of the participants. Measurements of height and weight of each participant were performed using a portable stadiometer (accuracy ± 0.05 cm) and a digital weighing scale (accuracy ± 0.25 kg), respectively.

Early morning midstream urine samples were collected from each participant (approximately between 5 and 7 a.m.) into sterile containers. Following collection, the samples were temporarily stored at 2–8 °C until the transit to the laboratory was undertaken. Urine samples were centrifuged at 1000× *g* for 15 min at 4 °C, and the isolated supernatants were stored at −80 °C until the analysis of biomarkers.

### 2.3. Assays for Biomarkers

The quantitative analysis of creatinine and albumin in urine samples was performed using a clinical biochemistry analyzer (HumaStar 100; Human GmbH, Wiesbaden, Germany), based on creatininase enzymatic and immunoturbidimetric methods, respectively. The minimum detection limits of the analyzer for urinary creatinine and albumin were 0.05 mg/dL and 0.01 mg/L, respectively.

Concentrations of KIM-1 and NGAL in urine samples were determined using enzyme-linked immunosorbent assay (ELISA) kits (Cusabio, www.cusabio.com; accessed on 20 October 2023) as per the manufacturer’s assay protocol. The intra-assay and inter-assay coefficients of variation (CVs) for the ELISA kits were <8% and <10%, respectively. Analysis of biospecimens was undertaken at the Biomarker Research Laboratory of the Department of Zoology, University of Ruhuna, Matara, Sri Lanka.

### 2.4. Data and Analyses

Absolute and creatinine-normalized urinary KIM-1, NGAL, and Cys-C concentrations deviated significantly from normality. Hence, a nonparametric statistical approach was adopted for data analysis and representations. Linear regression models were used for the exploration of associations between urinary creatinine levels and absolute and creatinine-normalized KIM-1, NGAL, and Cys-C concentrations. Statistical and data analyses were performed using IBM SPSS Statistics 26.0 (IBM Inc., New York, NY, USA), GraphPad Prism 9.4.0 (GraphPad Software LLC, San Diego, CA, USA), and Microsoft Excel 2021 (Microsoft Corp., Redmond, WA, USA).

## 3. Results

### 3.1. Participant Characteristics

Biomarker and urinary creatinine data of 2100 participants (1034 boys and 1066 girls) were analyzed for investigating associations between urinary creatinine concentration and biomarker levels. Baseline characteristics of the study participants are presented in Table 1. 

### 3.2. Associations of Urinary Biomarkers with Creatinine

**Figure 1:**
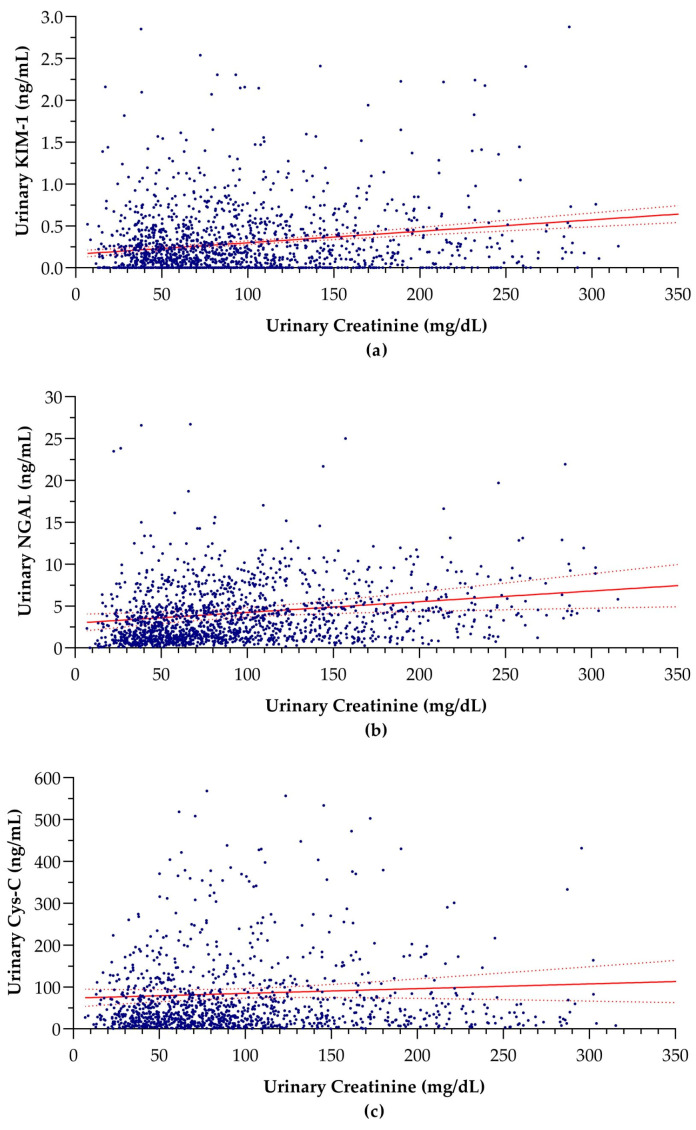
Linear associations between absolute biomarker concentration and creatinine concentration in urine: (**a**) KIM-1 (r = 0.171; *p* < 0.0001), (**b**) NGAL (r = 0.065; *p* = 0.009), and (**c**) Cys-C (r = 0.032; *p* = 0.250). The dashed lines surrounding the solid trend line indicate the 95% confidence intervals. KIM-1: kidney injury molecule-1, NGAL; neutrophil gelatinase-associated lipocalin, Cys-C: cystatin C, r = correlation coefficient, p = probability.

The associations of absolute concentrations of the three biomarkers with urinary creatinine concentrations are illustrated in Figure 1.

Urinary creatinine showed significant positive associations with absolute urinary KIM-1 (*p* < 0.0001) and NGAL (*p* = 0.009) concentrations, where no significant association was identified with urinary Cys-C (*p* = 0.250). The parameters of the linear regression models used in the analysis are given in Table 2.

As implied by the linear regression analysis, the significantly positive linear associations between creatinine and absolute KIM-1 and NGAL concentrations may provide a rationale for adjustment for urinary creatinine [13]. However, these associations are very weak and almost negligible. 

**Figure 2:**
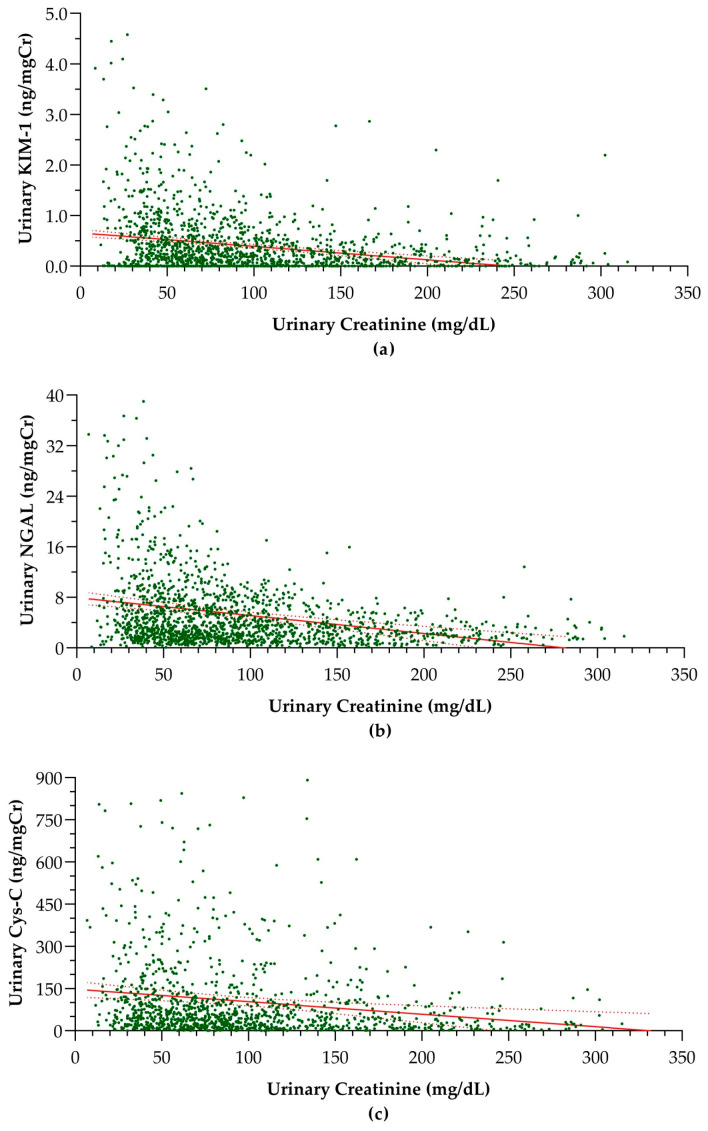
Linear associations between creatinine-adjusted biomarker concentration and creatinine concentration in urine: (**a**) KIM-1 (r = 0.039; *p* < 0.001), (**b**) NGAL (r = 0.148; *p* < 0.001), and (**c**) Cys-C (r = 0.099; *p* < 0.001). The dashed lines surrounding the solid trend line indicate the 95% confidence intervals. KIM-1: kidney injury molecule-1, NGAL; neutrophil gelatinase-associated lipocalin, Cys-C: cystatin C, r = correlation coefficient, p = probability.

Furthermore, creatinine-normalized KIM-1, NGAL, and Cys-C levels exhibited significant negative linear associations with urinary creatinine concentration (Figure 2).

The parameters of the linear regression models used in the analysis of associations between urinary creatinine and creatinine-normalized biomarker levels are presented in Table 3.

**Figure 3:**
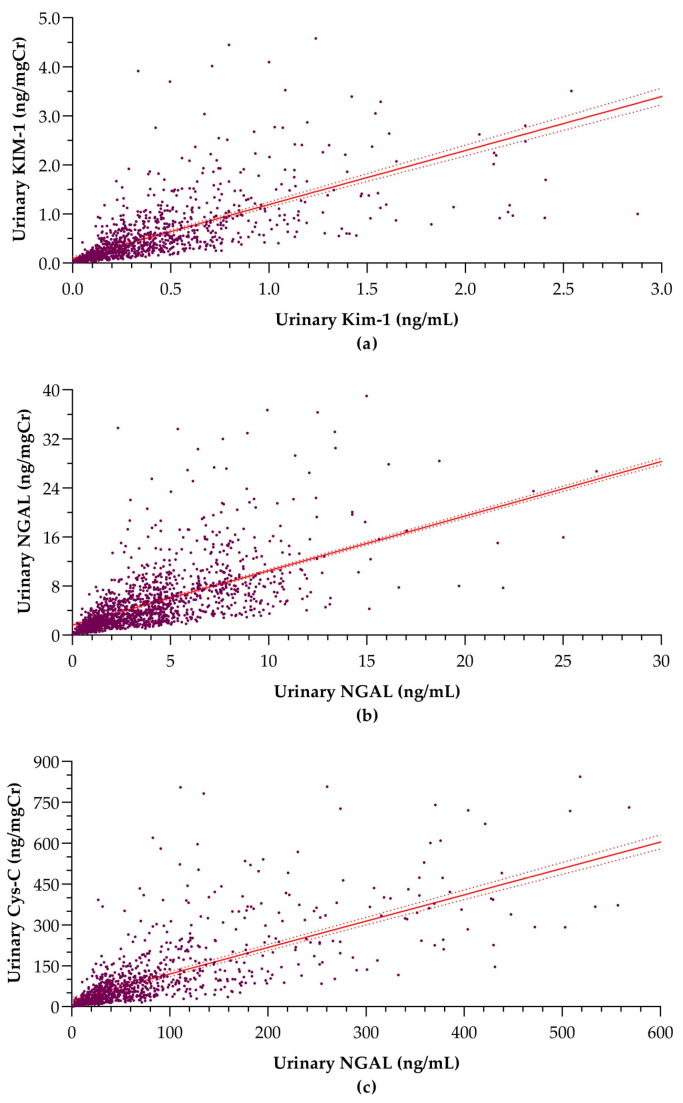
Linear associations between creatinine-adjusted biomarker concentration and absolute biomarker concentration in urine: (**a**) KIM-1 (r = 0.654; *p* < 0.0001), (**b**) NGAL (r = 0.918; *p* < 0.0001), and (**c**) Cys-C (r = 0.756; *p* < 0.0001). The dashed lines surrounding the solid trend line indicate the 95% confidence intervals.KIM-1: kidney injury molecule-1, NGAL; neutrophil gelatinase-associated lipocalin, Cys-C: cystatin C, r = correlation coefficient, *p* = probability.

These negative linear associations indicate over-adjustment of the biomarkers in their normalization to creatinine [13]. However, the correlations between urinary creatinine concentration and creatinine-adjusted biomarker levels are almost negligible (correlation coefficients < 0.2). Moreover, according to the linear regression models, the effect of urinary creatinine levels did not account for considerable variability in creatinine-adjusted biomarker levels. Supporting these weak negative linear associations, creatinine-adjusted urinary KIM-1, NGAL, and Cys-C concentrations also demonstrated strong correlations with corresponding absolute concentrations in urine (Figure 3).

Moreover, absolute concentrations of KIM-1, NGAL, and Cys-C showed significantly strong positive associations with their respective creatinine-normalized levels, as identified in the linear regression models (Table 4).

As implied by this analysis, adjustment to urinary creatinine was likely to result in no adverse variations in biomarker levels for this population, and creatinine-adjusted biomarker levels retained almost consistent variation as the absolute concentrations. Thus, with this rationale, both absolute and creatinine-adjusted concentrations of urinary KIM-1, NGAL, and Cys-C can be adopted equally well for the characterization of subclinical kidney injury in community screening for adolescents within the given age range.

## 4. Discussion

In the evaluation of kidney injury biomarkers, the consideration of normalization procedures is pivotal to ensure accurate and clinically meaningful interpretations of urinary biomarker concentrations. In this study, we explored the rationale for creatinine adjustment concerning three widely recognized biomarkers of kidney injury: KIM-1, NGAL, and cystatin C with 2100 children and adolescents enrolled in the C-KidnEES cohort in Sri Lanka. The absolute concentrations of KIM-1, NGAL, and ACR showed significant positive linear associations with urinary creatinine as identified in the regression analyses. These associations provide a potential rationale for adjustment of these biomarkers for urinary creatinine. However, this rationale is not strong enough to emphasize the need for creatinine adjustment, as the observed linear associations were weak. Furthermore, creatinine-adjusted levels of the three biomarkers resulted in significant but weak negative linear associations with urinary creatinine concentrations. This negative association is referred to as an “over-adjustment” to creatinine [13]. However, according to the linear regression models, this negative association was very weak, and the impact of over-adjustment appears almost negligible. Moreover, creatinine-adjusted biomarker levels showed a strong positive linear association with corresponding absolute biomarker levels. 

The production of creatinine and its urinary levels are variable depending on multiple factors including, age, sex, muscle mass, and exertion levels in individuals [14]. Moreover, creatinine is also subjected to tubular secretion during urine production. Hence, it is posited that adjustment to creatinine may not be a precise approach for normalization of tubular injury biomarkers to eliminate the effect of urine output, due to this variability in urinary creatinine levels [15]. However, the strong positive associations between absolute and creatinine-adjusted biomarker levels imply that adjustment to creatinine does not elicit an adverse effect on biomarker levels and retains the same consistent variation. Thus, the effect of possible under-adjustment or over-adjustment does not appear to be a significant erratic issue in the adjustment of absolute biomarker concentrations to urinary creatinine for the given age group. Hence, these implications do not provide a particular rationale to justify the need for the adjustment of urinary concentrations of the above three biomarkers. 

In particular, KIM-1, NGAL, and Cys-C are mostly adopted in clinical studies to investigate kidney health outcomes associated with environmental risk factors and for the identification of kidney health risks in children and adolescent communities. For these studies, some researchers have adopted absolute urinary biomarker concentrations [16,17,18], while the interpretations of some other studies are based on creatinine-adjusted biomarker levels [19,20,21]. In addition to community health screening, urinary biomarkers are extensively used in predicting different nephropathies, including AKI and renal scarring in different clinical settings. Urine biomarker concentrations normalized to urine creatinine may offer an advantage in AKI diagnosis and may improve the prediction of AKI severity. This is particularly true in those patients undergoing a significant number of intravascular infusions (such as ICU, cardiac, or abdominal surgery). For instance, in a study with 528 adult patients, creatinine-normalized urinary biomarkers (KIM-1, NGAL, and Cys-C) best predicted the development of AKI and dialysis, while absolute urinary biomarker concentrations diagnosed AKI best on admission [22]. According to these interpretations, creatinine adjustment of urinary biomarkers improves the prediction of developing AKI, but it does not appear advantageous in the diagnosis of established AKI. 

Theoretically, high urine biomarker values occur more frequently in subjects with concentrated urine, while patients with diluted urine are less likely to present high urine biomarker values [23], suggesting that the interpretation of urinary biomarkers, together with an assessment of whether urine is diluted or concentrated, can better discriminate between various degrees of AKI. However, after reviewing all of the important published manuscripts on NephroCheck (a cartridge-based commercial test system for the identification of AKI using biomarkers), the most promising urinary biomarkers of AKI were found to be tissue inhibitor of metalloproteinases-2 (TIMP-2) and insulin-like growth factor-binding protein 7 (IGFBP-7), although there were no clear differences between trends in NephroCheck (concentration measured or normalized to urinary creatinine excretion or urine osmolality) for AKI versus non-AKI [24,25]. Thus, the optimal quantitative approach for urinary biomarkers to characterize AKI appears to be dependent on the outcome of interest.

Moreover, studies have identified a potential role of urinary KIM-1, NGAL, and Cys-C in the assessment of renal scars in pediatric and adult subjects with vesicoureteral reflux (VUR). Significantly elevated creatinine-normalized urinary NGAL [26,27] and KIM-1 [28] and absolute urinary Cys-C [29] levels have been observed in UVR patients with renal scars compared to UVR patients with no renal scars. On the contrary, a few studies have reported no significant increases in creatinine-adjusted or absolute urinary KIM-1, NGAL [30], and Cys-C [31] in subjects with renal scars compared to those with no renal scars. In addition, significantly increased creatinine-normalized urinary NGAL, Cys-C [32], and KIM-1 [33] levels have been identified in subjects with urolithiasis, suggesting a potential role of these biomarkers in predicting associated renal injury. Furthermore, multiple studies have suggested urinary KIM-1, NGAL, and Cys-C as potential markers of diabetic nephropathy [34,35]. 

Concerning these studies, researchers have adopted both creatinine-normalized and absolute biomarker concentrations for diagnostic and predictive interpretations of kidney health outcomes in communities and diseased subjects. However, none of these studies have provided a particular rationale for the use of adjusted or absolute biomarker levels. Hence, it is important to note that the choice of normalization method and the interpretation of results should be made carefully and in consideration of the specific research question and the characteristics of the study population.

Creatinine in circulation is subjected to glomerular filtration, and an extent of 10–40% is secreted by the proximal tubule [36]. Hence, this adjustment is based on the assumption of normal glomerular filtration and tubular secretion with constant urinary creatinine excretion [37]. In this consideration, creatinine adjustment is more applicable for healthy individuals with normal kidney function. Moreover, studies with healthy children and adolescents have also demonstrated significant variations in urinary creatinine excretion with varying age and sex. In particular, an increase in urinary creatinine excretion was observed in girls and boys following puberty. Rapid growth with increased muscle mass is attributed to observed increases in urinary creatinine excretion [38,39]. Hence, these variations appear to result in significant deviations in urinary biomarker levels when adjusted to creatinine leading to potentially inaccurate interpretations. However, this may not provide a strong rationale for the use of absolute urinary biomarker concentrations, as it is also affected significantly by variations in urine output due to hydration status.

Impaired kidney function in patients and declined kidney performance in older individuals can result in significant abnormalities in creatinine excretion [15]. Studies with participants in different clinical settings have identified potential overestimations or underestimations of urinary biomarker levels (KIM-1, NGAL, and Cys-C) due to creatinine normalization. A study with hospitalized adult patients with changing glomerular filtration rates identified notable variations in urinary creatinine excretion within and across individuals. In particular, urinary kidney injury biomarkers in these patients gave more important indications on disease progression and therapeutic efficacy. Thus, the observed variability in creatinine excretion can interfere with such determinations and in setting up threshold values for biomarkers to distinguish diseased subjects from healthy subjects [37]. Moreover, the use of certain drugs such as cimetidine and trimethoprim is known to influence the tubular secretion of creatinine [9,40]. Creatinine production in muscles is also known to be interfered positively by rhabdomyolysis and negatively by severe hepatic diseases [7,41]. Thus, the influence of the above factors can result in significant changes in urinary creatinine concentrations, and in such cases, the adjustment of tubular markers to creatinine can be particularly misleading [15]. 

Due to these limitations of the creatinine-adjustment approach, the specific gravity of urine has also been suggested as an alternative. Specific gravity refers to the ratio between the density of urine and the density of pure water at the same temperature. However, this measure is significantly affected by hydration status, where low water intake results in low urine output with increased concentration and in turn, increased specific gravity. Moreover, proteinuria in kidney diseases also leads to higher specific gravity in urine. Thus, in these scenarios, biomarker levels adjusted to specific gravity tend to be lower, leading to inaccurate interpretations. However, for the studies that have compared individuals or communities with notable variability in muscle mass and meat intake, specific gravity normalization has been suggested to be more accurate than creatinine adjustment, due to the relatively higher dependence of urinary creatinine on the above factors [42].

As an alternative option, researchers suggest the collection of timed urine samples for a sufficient period through which the analyte remains stable for the estimation of actual excretion rates of biomarkers (e.g., 24 h urine sampling) for comparisons. This approach can greatly average out the effect of urine output variation in biomarker levels, but it can still result in missed samples and a high tendency for contamination. Moreover, this approach is feasible in hospital settings, but it becomes greatly impractical in community screening. Here, an overnight urine sample where the first morning urine sample is collected is considered more practical and convenient for timed urine sampling [43,44]. Finally, due to these limitations of the normalization approaches discussed above, some researchers have suggested the use of absolute biomarker concentrations with no adjustment [45,46].

Importantly, it is worth highlighting a few strengths of the present study. Mainly, here we maintained a large sample size including 2100 girls and boys within the age range of 10–18 years for the assessment of the validity of creatinine normalization for three widely used renal biomarkers in community studies. This study encompasses a favorably broad age range in which the children and adolescents showed rapid growth for making interpretations on biomarker adjustment with high variability in urinary creatinine. Apart from these key strengths, there exist a few limitations also. Mainly, these findings may not be equally applicable for the whole pediatric age range nor for the other biomarkers of kidney injury. Moreover, in the present study, we analyzed the first early morning urine samples of the subjects resembling a timed urine sampling. Thus, the urine samples were not likely to be affected by certain confounding factors such as physical exertion. However, concerning spot urine samples collected during the daytime, the effects of physical exertion and time of the day may be different for creatinine excretion rates in individuals [8,10]. Hence, the findings of this study may not be precisely applicable for making decisions on the creatinine adjustment for spot urine samples of children and adolescents. Moreover, in the present study, we recruited healthy school students in the age range of 10–18 years to study urinary biomarkers of kidney injury in relation to urine concentration. However, the expression of these biomarkers in children and adolescents may exhibit different health scenarios. Thus, the applicability of creatinine adjustment for kidney injury biomarkers in subjects with diverse diseases in different age groups must be studied in depth. Furthermore, verification of the accuracy and precision of the use of absolute renal injury biomarker concentrations in place of creatinine-adjusted biomarker levels also contributes to cost-effective diagnostic approaches in mass screening for kidney diseases. 

## 5. Conclusions

Within the spectrum of the findings of the present study, normalization of urinary KIM-1, NGAL, and Cys-C to urinary creatinine does not result in significant under-adjustment or over-adjustment to absolute urinary concentrations, giving no specific rationale for creatinine adjustment. Hence, absolute urinary concentrations of the above biomarkers can be adopted for characterization of subclinical kidney injury in adolescents in community studies where early morning urine sampling is practiced. However, in the studies with spot urine sampling, consideration of both absolute and creatinine-normalized biomarker levels would be a better approach depending on the study settings.

## Figures and Tables

**Table 1:** Baseline characteristics of the study participants.

Parameter	Value
Age (years)	14.27 (13.15–15.29)
BMI (kg/m^2^)	17.44 (15.51–19.76)
UCr (mg/dL)	76.690 (49.365–119.012)
Creatinine-adjusted urinary biomarker levels	
KIM-1 (ng/mgCr)	0.165 (0.003–0.488)
NGAL (ng/mgCr)	3.241 (1.659–6.364)
Cys-C (ng/mgCr)	48.501 (18.921–112.106)
Absolute urinary biomarker levels	
KIM-1 (ng/mgCr)	0.143 (0.001–0.387)
NGAL (ng/mgCr)	2.826 (1.241–4.975)
Cys-C (ng/mgCr)	36.857 (15.460–83.853)

Values are given as the median (interquartile range).

**Table 2:** Associations of urinary creatinine with absolute concentrations of injury biomarkers, in linear regression models.

Biomarker	B	95% CI of B		β	t	*p*
KIM-1	0.001	0.001	0.002	0.171	7.031	<0.001
NGAL	0.013	0.003	0.022	0.065	2.620	0.009
Cys-C	0.115	0.081	0.310	0.032	1.152	0.250

Independent variable: urinary creatinine concentration. The linear regression models include the following: KIM-1, y = 0.162 + 0.001x (R^2^ (adjusted) = 0.0029, *p* < 0.001); NGAL, y = 2.962 + 0.013x (R^2^ (adjusted) = 0.004, *p* =0.009); and Cys-C, y = 73.520 + 0.115x (R^2^ (adjusted) = 0.001, *p* < 0.25).

**Table 3:** Associations of urinary creatinine with creatinine-adjusted renal biomarker levels, in univariate linear regression models.

Biomarker	B	95% CI of B		β	t	*p*
KIM-1	−0.003	−0.003	−0.002	−0.198	−8.182	<0.001
NGAL	−0.028	−0.038	−0.019	−0.148	−6.060	<0.001
Cys-C	−0.451	−0.704	−0.198	−0.099	−3.493	<0.001

Independent variable: urinary creatinine concentration. The linear regression models include the following: KIM-1, y = 0.658 − 0.003x (R^2^ (adjusted) = 0.039, *p* < 0.001); NGAL, y = 7.948 − 0.028x (R^2^ (adjusted) = 0.022, *p* < 0.001); and Cys-C, y = 147.274 − 0.451x (R^2^ (adjusted) = 0.009, *p* < 0.001).

**Table 4:** Associations between absolute and creatinine-adjusted urinary biomarker levels, in univariate linear regression models.

Biomarker	B	95% CI of B		β	t	*p*
KIM-1	1.101	1.039	1.162	0.654	35.037	<0.001
NGAL	0.890	0.871	0.871	0.918	94.024	<0.001
Cys-C	0.975	0.928	0.928	0.756	40.510	<0.001

Independent variable: absolute concentration of the respective biomarker in urine. The univariate linear regression models include the following: KIM-1, y = 0.092 + 1.104x (R^2^ (adjusted) = 0.428, *p* < 0.001); NGAL, y = 1.632 + 0.890x (R^2^ (adjusted) = 0.844, *p* < 0.001); and Cys-C, y = 23.18 + 0.957x (R^2^ (adjusted) = 0.571, *p* < 0.001).

## Data Availability

The datasets generated and analyzed during the current study are not publicly available due to restrictions under the approval of the ethics review board. Still, they are available from the corresponding author upon reasonable request.
